# Interviewing to develop Patient-Reported Outcome (PRO) measures for clinical research: eliciting patients’ experience

**DOI:** 10.1186/1477-7525-12-15

**Published:** 2014-02-05

**Authors:** Anne Brédart, Alexia Marrel, Linda Abetz-Webb, Kathy Lasch, Catherine Acquadro

**Affiliations:** 1 Psycho-Oncology Unit, Institut Curie, Paris France and Psychopathology and Health Process Laboratory (EA 4057), Psychology Institute, University Paris Descartes, 26 rue d’Ulm, 75246 Paris, France; 2 Mapi, HEOR & Strategic Market Access, 27 rue de la Villette, 69003 Lyon, France; 3 Patient-Centred Outcomes Assessments, Bollington, Macclesfield, Cheshire, UK; 4 Pharmerit International, 245 First Street, Suite 1800 18th Floor, 02142 Cambridge, MA, USA; 5 Mapi Research Trust, 27 rue de la Villette, 69003 Lyon, France

**Keywords:** Patient-reported outcome measures, Qualitative research, In-depth, Semi-structured, Structured interviews, Focus group, Cognitive interviews, Interpersonal, Communication skills

## Abstract

Patient-reported outcome (PRO) measures must provide evidence that their development followed a rigorous process for ensuring their content validity. To this end, the collection of data is performed through qualitative interviews that allow for the elicitation of in-depth spontaneous reports of the patients’ experiences with their condition and/or its treatment. This paper provides a review of qualitative research applied to PRO measure development. A clear definition of what is a qualitative research interview is given as well as information about the form and content of qualitative interviews required for developing PRO measures. Particular attention is paid to the description of interviewing approaches (e.g., semi-structured and in-depth interviews, individual vs. focus group interviews). Information about how to get prepared for a qualitative interview is provided with the description of how to develop discussion guides for exploratory or cognitive interviews. Interviewing patients to obtain knowledge regarding their illness experience requires interpersonal and communication skills to facilitate patients’ expression. Those skills are described in details, as well as the skills needed to facilitate focus groups and to interview children, adolescents and the elderly. Special attention is also given to quality assurance and interview training. The paper ends on ethical considerations since interviewing for the development of PROs is performed in a context of illness and vulnerability. Therefore, it is all the more important that, in addition to soliciting informed consent, respectful interactions be ensured throughout the interview process.

## Introduction

Medical advances, increasing specialisation, rising patient expectations, and the complexity of health care have expanded the range of clinical research questions to specifically address the patient’s experience of illness and treatment [1]. The importance of addressing patients’ perspectives to assess the value of medical interventions is currently widely recognised, particularly in situations where only the patient has direct knowledge of treatment benefit or where no agreed upon biomarkers exist [2-4].

Patients’ perspectives on the symptoms they experience, how they feel and function, and their quality of life associated with their health condition and its treatment may be measured with patient-reported outcome (PRO) measures. A PRO measure provides a report on the status of a patient’s health condition that comes directly from the patient, without interpretation of the patient’s response by the clinician or anyone else [5].

PRO instruments may be used in different clinical contexts, such as: 1) clinical trials (medical product development - for assessing treatment benefits or support labelling claims from the patients’ perspective –clinical endpoints for regulatory submission), 2) “real world” studies (e.g., market research, effectiveness studies, public health research) to assess patients’ health care needs, medical product acceptability, preference, adherence and related factors (barriers or facilitators) or 3) clinical practice (e.g., to screen; identify and monitor patients’ symptoms, difficulties, and health care needs, and to support shared medical decision making).

Whereas quantitative research measures results using numerical data and answers questions of “how many/how much?” qualitative research answers questions such as *“what is X, and how does X vary in different circumstances, and why?”’* rather than *“how big is X or how many X’s are there?”*[6], and gathers verbal or observational data [7].

Although qualitative research has long made contributions in the health care field, in recent years, several researchers stressed the importance of this approach as the necessary starting point for developing measurement tools to be used in quantitative research in order to ensure content validity [8-12]. Content validity, measuring what one intends to measure, is the measurement property in this context that assesses whether items are comprehensive, well understood and adequately reflect the patient perspective from the population of interest [8-14].

Qualitative research is an umbrella term for various theoretical models and data collection methods [15]. Among these, an over-arching phenomenological approach with grounded data collection and analysis methods has been suggested to most accurately include the patient’s voice in PRO development [8]. Indeed phenomenological research is designed to discover and understand the meaning of human life experiences [16]. Such an approach which addresses the question, “What is it like to have a certain experience?” is particularly suited to explore the patient’s perspective and study a condition through the eyes of the person living with it [8].

Recent papers addressing the rigorous use of qualitative research approaches in the development of PROs have focused on the procedures of data collection and analysis in terms of sampling (representative experiences, saturation), type of data collected (lived experiences explored from sensitizing concepts), mode of analysis (coded meaning units, categorized and structured), and results (conceptual framework, patients’ own words) [8,9,11,12]. Key attributes of scientific research, validity, and reliability in this context have been highlighted. What has not been addressed sufficiently in this literature is how best practices in interviewing methods can contribute to the quality and usefulness of the data collected. During the data collection phase, the qualitative approach and method should be appropriate to the research issue and the information collected should provide sufficient details and depth to provide insight into the meaning and perceptions of informants [17].

This paper provides a review of qualitative research applied to PRO measure development with a clear definition of what is a qualitative research interview as well as information about the form and content of qualitative interviews required for developing PRO measures. Particular attention will be paid to the description of interviewing approaches. Information about how to get prepared for a qualitative interview will be provided with the description of how to develop discussion guides for exploratory or cognitive interviews. Interpersonal and communication skills required to facilitate patients’ expression will be described in details, as well as those needed to facilitate focus groups and to interview children, adolescents and the elderly. Special attention will be also given to quality assurance and interview training. The paper will end on ethical considerations since interviewing for the development of PROs is performed in a context of illness and vulnerability.

## Review

### What is a qualitative research interview?

The qualitative research interview may be defined as a scientific research process based on verbal communication aimed at gathering information in relation to a specific aim [18]. Unlike a reciprocal conversation, often the topic in a qualitative interview is controlled by the interviewer who seeks information from an interviewee, a person who has had the relevant experience, but the interviewee has considerable freedom to respond to open-ended, but focused, questions [19].

In the present context, i.e., the development of PROs, the interviewee is mainly a person who is affected by a disease; so in the text below, the word patient often refers to the interviewee. Qualitative interviews, however, may also be performed with clinicians or caregivers either as experts helping to delineate the patient-related concepts to address or as subjects for whom subjective rating scale instruments, e.g., clinician-reported outcome (ClinRO) or observer-reported outcome (ObsRO) measures, may also be developed.

This paper will focus on interviews to develop PRO measures and only briefly describe issues involved in the development of ClinROs or ObsROs and interviews with special populations, e.g., children.

### Forms of qualitative research interviews

#### a. Depth, standardisation, interviewer control

Various interviewing approaches exist depending on the depth of the interview, the standardisation of the discussion guide and how structured the interviewer wishes the interview to be [20-22]. In quantitative or survey research, interviews are structured and standardised, asking the same set of specific close-ended questions in precisely the same way to every participant. In contrast, the in-depth unstructured or semi-structured interview, used in qualitative research, rests on open-ended questions relatively formulated or ordered in a standardised way, developed to elicit spontaneous, non-biased reports from the patient [20,21]. The in-depth interview raises open-ended questions to allow further questioning based on the patient’s responses. In-depth interviews explore a condition in detail and seek to unravel and clarify the complex problems directly and indirectly associated with a condition and their inter-relationships [23].

Focusing on subjectivity and experiential data, i.e., how individuals perceive and understand their feelings, sensations or actions, the in-depth interview is particularly appropriate to identify issues of interest for developing questionnaires [8]. The semi-structured interview is also useful at a later stage of the questionnaire development, namely to address patients’ understanding of questionnaire items and check for content validity [24].

Table 1 displays the different methods of inquiry in open- versus closed-ended interview questions. Open-ended questions (e.g., “Tell me about your experiences with surgery,” allow for an in-depth and extended personal account expressed in the individual’s own words. Closed-ended questions elicit precise specific confirming/disconfirming information, and unless previously suggested by the patients, may potentially bias their responses (e.g., “How worried are you about the results of your surgery? Would you say, ‘not worried at all’ to ‘extremely worried’?”).

**Table 1:** Differences between open-ended and closed questions

***Open-ended questions***	***Closed questions***
In-depth interviews	Standardized interviews
E.g., “What is it like for you to have X (insert condition)?”	“How would you rate your (insert specific concept, such as pain) on this 0-100 scale?
Describe an average day, living with X (insert condition). How about a good day? A bad day?	Do you have (insert specific concept)?
*Allow for responding in one’s own words*	*All persons answer the same questions allowing for comparisons*
*Do not suggest answers*	*Less variable answers*
*No a priori framework, inductive approach*	*Deductive approach, allows for quantification*
*Necessary to develop closed questions to probe*	
*May help understand deviant answers to closed questions – tell me more about that*	

Adapted from Boutin [18], p119.

In contrast to the quantitative research paradigm where hypotheses are raised and a deductive approach performed, in qualitative research, preconceived ideas have to be abandoned [25]. The interviewer’s attitude must be open in the dialogue in which he/she invites the subjects. Hence, the qualitative research interview follows primarily an inductive approach; data-gathering is subject to ongoing revision; interview questions are flexible and individualised, and the emphasis is placed on depth of information [26].

Interviews can be more or less directive [20]. In non-directive interviews, the interviewer is a catalyst who only guides the subject on pre-set investigation themes by probing for clarification, reformulating or reflecting his/her understanding of what the patient has said [18]. Interviews in the context of PRO questionnaire development use this non-directive approach.

#### b. Individual or group interview (focus group)

Qualitative interviews may be conducted with one or few individuals. Both individual and group interviews may be viewed as complementary, providing different perspectives on patients’ viewpoint.

In individual interviews, the interviewee and the interviewer co-construct a discourse that is mostly spoken by the interviewee, but also contains the interviewers’ expectations through interjections or questions [22]. In contrast, group interviews or focus groups are “social spaces in which participants co-construct the ‘patient’s view’ by sharing, contesting and acquiring knowledge” [27].

An individual interview usually allows for a more private environment to facilitate the sharing of more in-depth information, and to help the interviewee feel more comfortable discussing potentially sensitive/embarrassing questions [28]. Group interviews (focus groups) allow the collection of data on more individuals; and are particularly useful in uncovering why participants think, act, or feel as they do [29]. During group discussion, participants agree or disagree with each other; explain their perspectives providing insight into factors that lead to endorsing ideas. However, focus groups also present limitations: participants may give socially desirable responses, i.e., create a consensus rather than generate ideas [9]; perception of self and disease may be altered by group participants; and it may be difficult to go in depth with the subjects [28]. In focus groups, the facilitator needs to ensure that all participants have an opportunity to partake and that no one person dominates the discussion. This can be extremely challenging and therefore requires a very experienced facilitator.

Focus groups may be employed at the very start of a new project to select major questions for the discussion guide [26] or at a later confirmatory phase aimed at assessing the preliminary conceptual model, the topics selected and their interrelationship. Focus groups are generally an inappropriate method for questionnaire pre-testing in which detailed assessment of items’ wording is performed, given that participants may be reluctant to admit in public that they have difficulty understanding something [24].

In the focus group setting, care needs to be taken that severity levels are not intermingled, as this can have negative consequences on those who ‘see what is coming’ (particularly in progressive diseases) or for those who are seen as severe and outside the realm of the others. Severity level, age, or gender-specific focus groups can be conducted to ensure an appropriate spectrum of patients and facilitate discussion.

### Preparing for the qualitative research interview

Good preparation for the qualitative interview facilitates a sound methodological plan. It can increase confidence and permit concentration on what the person is saying [19]. This requires the development of a discussion guide: a general plan of questions to ask, highlighting the specific important themes to address [8,9]. This preparation allows for thinking through how to word open-ended questions and helps to avoid the formulation of loaded questions that bias responses [19]. For example, instead of using the word “problem” which may bias towards problems, a more useful approach is to ask to describe one’s illness.

The discussion guide determines a focus for the inquiry [18], establishes boundaries, although flexible, for the study, and provides inclusion/exclusion criteria for new information. It also determines the successive phases of the inquiry with less and more focus [8]. It is developed in an iterative process through several stages guided by broad concepts [26].

Referring to the above-mentioned interviewer control, it should be noted that if there is a guide, the guide is meant as a guide, not a script. The interviewer should know the guide well and allow the interviewee to guide the discussion, while the interviewer probes or asks relevant questions at the time that the interviewee brings them up, rather than waiting until that point in the guide. That will allow a better flow of the conversation, but this relies on the interviewer understanding well the key objectives of the interview and requires that no key elements of the guide are missed.

#### a. Discussion guide for an exploratory interview

At the initial stage of a PRO development, patients’ interviews are exploratory. The purpose is to highlight the domains or sub-domains of patients’ specific experience and to provide a conceptual model on which to develop the instrument.

A discussion guide may use concepts from already-used instruments, other qualitative literature exploring the same phenomenon, acquired knowledge of the clinical condition, or interviews with clinicians [8]. These concepts, qualified as *sensitizing*, are initial ideas that are used to collect focused data and to clarify the data [19,30].

For exploratory interviews, the number of questions is relatively small to allow for probing into details of the patient’s experience.

An interview guide includes 2 or 3 stages [22,28]: the starting instruction, the body of the interview and the conclusion.

The starting instruction gives direction to the content of the interview, constrains the discourse on this theme, and frees the speech of the interviewee by assuring confidentiality to him or her. This stage establishes the relationship between the interviewer and the participant and often collects biographical details. The interviewee is at once informed that there are no right or wrong questions [28].

The body of the interview can include questions that are more or less structured, whether the interview is semi-structured or non-structured. During this stage, the interviewer begins with more open-ended questions (e.g., “Tell me about when you first received the diagnosis of…”) and then moves progressively to more personal and sensitive questions using more direct questions about a specific concept of interest, but still in an open ended manner (e.g., for a disease where sexual dysfunction is a main component of the research question - “You mentioned earlier that your intimate relationship with your spouse has been affected by your condition, can you tell me more about that?…”), using the pre-planned topic guide where the line of questioning is guided by the interviewee’s responses and the interviewer adopts a receptive approach (e.g., “Can you tell me more about that?”). Sometimes, allowing the person to gain some distance from their condition can provide additional responses not brought up in traditional questioning. Asking the patient to do ‘homework’ prior to the interview, in the form of putting together a collage or drawing that describes their experience of their condition, can immediately open the interview with useful and very pertinent and emotive information. Drawings, for example, may be particularly helpful for facilitating children or adolescents’ interviews [31].

The last stage includes the conclusion with final closing questions (“Is there anything else that you feel is important that we haven’t discussed yet?”). The end of the interview is particularly important for this is often at this time that ideas emerge which may give explanations on the overall narrative; it is also important to provide closing questions and avoid an abrupt end. Charmaz [19] emphasizes the need to end interviews on a positive tone.

#### b. Discussion guide for cognitive interviews

At a subsequent stage of PRO development, when a preliminary version of a conceptual model is established, the purpose of the interviews is to have patients confirm the relevance of the concepts selected. A final version of the conceptual framework is then elaborated to develop items assessing the relevant concepts [32].

After the concept elicitation interview data is analysed, a preliminary instrument is developed. Cognitive interviews are then conducted to verify the relevance and comprehension of the instrument to the population of interest. This phase assesses whether items (item stem and response options), questionnaire instructions, and recall period are understood by patients as intended by the item designer, i.e., in accordance with the underlying concept. This is evaluated by analysing the process of answering questionnaire items, identifying how and when an item does not achieve its objective [24,33].

Whereas interviews addressing the patient’s experience of illness may have a more emotional tone, interviews aimed at addressing comprehension have primarily a cognitive focus. Cognitive interviews are carried out using two specific techniques: the think aloud technique, whereby patients are invited to express out loud their thought process while reading and answering the item; or the probe technique which essentially asks the patient to paraphrase items [34-36]. Probes address the meaning of the question for the patient (e.g., meaning of words, concepts, item frame of reference); the relevant information patients are retrieving from memory to answer the item; the thought process implied in formulating his/her answer (recalling, formulating or reporting answer); or further thoughts/interpretations that are elicited by the item.

### Interviewing skills and environment

The location of the interview may impact the interviewee’s discourse. It is important that a safe and comfortable environment is provided so that the interviewee feels comfortable. An institutional place like a hospital, clinic or medical office may induce a more passive position of the interviewee, but may be more comfortable and convenient than a neutral place. When the interview takes place in the interviewee’s home, the interview can be disturbed by day to day activities and the interviewee may be reluctant to speak freely in front of family members. Care also needs to be taken for the safety of the interviewer when making home visits. A neutral place like a centralized location, a hotel conference room or a research facility is often the optimal place.

#### a. Interpersonal skills

Throughout the interview, it is important to allow plenty of time, stay calm, be patient and not hurry [37]. The researcher must develop a positive relationship with the interviewee [20,21], allowing for equality, trust and involvement [38]. This requires fundamental qualities at both the human/interpersonal and technical/communicational level.

Notions of appropriate interpersonal skills for conducting a qualitative research interview may be drawn from the field of counselling and psychotherapy. According to Rogers [39] a trusting listening environment is characterized by the following conditions: *authenticity or congruence, unconditional positive attention and empathy*. So, in order to facilitate the patient being spontaneous and open, the interviewer should be *him/herself* and not play a “role”. Situations where the interviewee is wondering (“what do you want from me?” or “how do I have to answer to meet your expectations?”) suggest that the interaction is not working well, which may bias the collection of interview data.

Further, the interviewee should be allowed free expression without being judged or negatively appraised. One should keep in mind that interviewing is meant to explore and not to interrogate [19]. The interviewee’s spontaneity may be affected by his/her representations of the researchers’ affiliations and sponsors. The interviewer should manifest interest, concern and empathy, i.e., understanding the subject as a person by immersing oneself in his subjective world.

More generally, the interviewer should avoid a “buddy” relationship with the interviewee, refraining from using terms too familiar or specific to the interviewee’s community. Creating a too friendly, therapeutic, educational or inquisitional relationship may induce inappropriate interactions (e.g., the interviewee’s expectations of the interviewer expressing his/her own experience, offering help, information or advices, or negative judgments) [18]. In particular, the qualitative research interview is neither a clinical nor a counselling interview. Rather than receiving therapeutic help, the patient offers help in providing his/her insights.

Interviewed on their experience of illness, patients sometimes tell painful stories; Charmaz [19] advises to just listen and try to understand the experience through the patient’s eyes and to validate its significance for him/her. The simple act of having a tissue box available and offering a tissue if needed often provides additional empathy and understanding at difficult moments and can get the discussion back on track quickly.

It is important to note that, due to regulatory obligations, interviewers sponsored by the pharmaceutical industry to develop a PRO, may be obliged to report any sponsor-product adverse events reported to them during the interview to the sponsor. In such cases, it is recommended that the interviewer ask the patient for permission to report the information to the company (and this is usually included in the patient consent form). This can, however, impact on the flow of the conversation, and therefore it is recommended to come back to this issue at the end of the interview.

#### b. Communication skills

Interviewing encompasses non-verbal and verbal communication aspects of which one needs to be aware. Particular attention should be given to non-verbal aspects like the interviewer’s and the interviewee’s physical attitude, including body movement and posture, gestures, facial expressions like smiles, gaze or voice tone. These may help to assess the quality of the interaction and allow for identifying that the interaction is not going well and has to be managed alternatively [18].

As described in Table 2, specific listening skills may be useful, such as active listening; allowing silence; reflecting; synthesizing; recognizing resistance [18,20,21,35,40-43]. These skills are competencies that may be learned.

**Table 2:** Listening skills

**Listening skills**	**Definitions**
Active listening	Listening with attention to the interviewee’s speech; participating actively with probes to enable going further.
Attentive silences	Differentiating between heavy silence (after an intrusive question), silence allowing to take breath, silence to reflect upon the question, silence in the rhythm of the interview.
Reflecting	Reflecting back the information may encourage further disclosure.
Synthesizing	Helps to check understanding of the interviewee before addressing another topic; it gives the interviewee the opportunity to correct if there is misunderstanding; it also indicates that the interviewee’s narratives has been heard.
Recognizing resistance	In face of avoidance, unauthentic testimony, reflect on what happens, underline that there is no right/wrong answer, rehearse aims of the research interview.

Active listening is a specific communication skill, based on the work of Carl Rogers [39]. The interviewee is given space to tell his story in his own way and the interviewer shows his/her understanding and acceptance [43].

Active listening also includes the skills for recognising and exploring patients’ cues, like the expression of concerns or the attempt to explain symptoms [40]. Specific verbal communication techniques appropriately help to explore the interviewee’s experience. Relevant questioning or clarifying (probing) is performed with an attitude that follows closely the interviewee’s discourse and provides latitude for expressing ideas. Clarifying questions helps to pursue ideas, offering necessary details or comments. These probing techniques require being able to elicit the right information, to drive the word flow, to choose between open or directive questions, and to attend to cues and explore the underlying experience.

Table 3 provides examples of eliciting questions.

**Table 3:** Examples of eliciting skills

**Eliciting skills**	**Examples**
Descriptive questions	● “Grand Tour” – placing the interview in context
	- What’s a typical day like for you?
	● “Mini Tour” – focusing on smaller unit of experience
	- Can you tell me more about that?
Checking	● Are there different kinds of bad times?
	● Are there different kinds of bad days?
	● Is this how you would talk about bad days to your friends? Your parents? Your doctor?
Contrast questions	● Investigates types of experiences and how they vary by context
	- You said that you get a lot of backaches, what, if anything, makes the experience of one of your backaches different from other aches and pains?

Prompting, repeating, rephrasing or checking are proposed according to the interviewee’s comments. In doing so the interviewer has to rely on his/her own experience, imagination and intelligence while keeping the interview’s objectives in mind, i.e., allowing the patient’s discussion of his/her experience.

See below an example of interview on the consequence of fibromyalgia on daily life:

Interviewer: What did fibromyalgia change in your life, if anything?

Patient: Many things.

Interviewer: Can you tell me more about that?

Patient: … regarding pain…(long silence)

Interviewer: … regarding pain?…[repeating, long pause (indicates a significant pause after which the interviewer prompts)]

Patient: … we are limited in doing things… how could you explain that, everyday life… at work…

Interviewer: so, pain change things at work… in everyday life (reflecting)… could you give me examples of what has changed at work? How about in everyday life? (clarifying)

#### c. Specific skills for facilitating focus groups

Focus groups require specific skills from the interviewer/facilitator-moderator [44,45]. The moderator has to quickly and acutely grasp the group dynamic (presence of leaders or shy, reserved individuals), facilitating a trusting relationship between participants and avoid boredom. He/she should not behave like a participant by sharing his/her opinions. Attention should be given to the moderator’s body language which can cue participants to what moderator thinks about what is said in the group. However, he/she may adopt an interventionist style [46], encouraging people to talk to one another.

As in individual interviews, active listening and probing skills are applied. Group participants’ discussion should be kept on track and the facilitator should decide when enough has been said on a topic. Along the group process, the facilitator-moderator has to listen (i.e., understand the meaning of what is said), think (i.e., appraise the breath and depth of group participant statements regarding the subject of interest) and manage the group simultaneously (i.e., involve shy or inattentive participants; interrupt dominant or disruptive participants).

Closure takes place by asking about missing information and thanking participants for their attendance.

#### d. Specific skills for interviewing children/adolescents or elderly

##### Children and adolescents

Interviewing children or adolescents requires knowledge of their specific cognitive, linguistic, affective, social and moral developmental characteristics in order to better understand their perspective [18]. Asking parents, in advance and out of earshot of the child, about words they use for their condition will make it easier to communicate with a child. Having the insight in advance of key words the children use will allow the interviewer to quickly connect with the child.

Simple, precise questions should be asked; to ensure that the child addresses the areas of key concern quickly. Care must be taken to use age appropriate language throughout the interview. As their attention may not span beyond 30 minutes, using play or drawings with children often facilitate expression and can break up the interview with fun activities that still provide useful information for the project objectives [31,47].

The relational aspects are important: the adult interviewer may be perceived differently depending on the child’s or adolescent’s perception of adult authority [48]. If perceived as an inquisitor, the motivation to authentically participate in the interview may be affected. However, if the interview is too abstract and indirect, this may raise insecurity in some children, or may lead to long, involved stories that have nothing to do with the research question – taking time and tiring the child to such a degree that it is then too late to get the answers to the research question. Children are very good at figuring out what an adult wants to know as this is what they are trained to do on a daily basis in school. It is extremely important to ensure that the child understands that the interview is not a test and that the interviewer is interested in what they think.

Autonomy needs of adolescents should be acknowledged. The interviewer should be prepared for adolescents’ desire to shock the interviewer. In such cases it is extremely important to maintain a non-judgmental response and to calmly query the statement if relevant or to move on to the next topic if the adolescent’s comment is not relevant to the research. For both children and adolescents; they should be ensured of confidentiality and be asked for their agreement to participate in the interview [18]. However, if a concern is raised during an interview that suggests a child is in danger, in many countries a ‘duty of care’ is required by ethics committees and it may be necessary to inform the child’s doctor or parent. As in the case of adverse event reporting, wording to this effect should be included in the parent consent form and the child assent form.

Providing children with an overview of what is included in the interview and how much time it will take is also an important technique. In addition, children should be provided with encouragement throughout the interview – ‘you’re helping me a lot; we are halfway done now’ or ‘only a few more questions to go’ – so they know how long they need to maintain their attention.

##### Elderly

Interviewing elderly patients also requires specific attention. Stereotypes (e.g., cognitive impairment), or a compassionate or too friendly tone may affect the interaction. Active listening to the elderly perspective through simple and direct questions may be helpful [18]. Interviewees who have difficulties providing detailed accounts of their experience, like the frail elderly, pose several challenges to the interviewer [37].

Frail elderly often tire easily, are less able to provide detailed descriptions due to sensory problems or present problems concentrating, leading to “thin” interview data. Difficulties with hearing can also slow down the interview process and potentially affect the relationship between interviewer and interviewee (given the need to ‘shout’ questions in such a situation). A quiet environment is absolutely essential for this population.

Qualitative research performed with frail elderly is threatened by focusing on a biased sample including patients that are less affected by the aforementioned problems. Strategies to maximise quality interviewing with this population include considering that thin descriptions may provide important information which should be corroborated with insights from more articulate participants. Specific interviewing strategies may be implemented such as giving more time to establish rapport, avoiding the patient’s disabilities to be unnecessarily exposed, establishing common understanding (words, concepts), offering extra support to narrate their experience and using cues like photographs, craft, and skills [37].

### Quality assurance and interview training

Interviewing is a method for data collection and criteria to evaluate the quality of this method include providing sufficient detail on: 1) whether the way data were collected addressed the research issue (appropriateness of the qualitative research method) and 2) whether the information were collected with sufficient details and depth to provide insight into the meaning and perceptions of the informants (adequacy of implementation of the qualitative research method) [17].

The adequacy of the qualitative interview implementation may be checked from the discussion guide, interview transcripts or excerpts/quotes. Interviews for PRO development are recorded and thus the suitability and sufficiency of the data collected may be assessed through transcribed data. Specific excerpts of interviews or interview quotes should provide evidence for the depth and details of interview content as well as on the accuracy of data interpretation and reporting.

These data must demonstrate richness, relevance and substance to allow for quality and credibility [19]. As also stated by Kvale [49], the data collected are expected to demonstrate maximum variation, which means that outliers are included when sampling so that, for example, all levels of severity are represented.

Few articles offer guidance for obtaining rich interview data; however, in a recent study, Ogden & Cornwell [50] evaluated the role of topic, interviewee’s characteristics and type of questions in predicting rich interview data. They found that open questions located later on in the interview and framed in the present or past tense rather than closed questions, located at the beginning or middle in the interview or framed in the future tense, provided more richness in interviewees’ answers. The preparation of interview guides should take this information into account.

To ensure high quality interviews, specific training should be implemented. Interviewing should be trained by modelling, discussion and rehearsing or role playing [42]. However, interviewing is also a craft [49] which requires skills that may be improved by repeated and adequate practice. For example, active listening is most effectively learnt experientially [43]. In addition, interviewers should understand that the interview narratives should not be contaminated by their pre-understanding, i.e., the meaning attributed according to personal experiences, theoretical understanding, hypotheses or assumptions [49].

This requires “reflexivity” which is defined as “an effort to reflect on how the researcher is located in a particular social, political, cultural and linguistic context” and allows a deeper sense of self as having multiple identities [25]. In other words, the interviewer should be aware of the influence necessarily introduced by his/her subjectivity or identity, and the way the discourse was co-constructed during the interview. This influence may be related to issues of interviewer-interviewee gender or to the interviewer’s role presentation (e.g., researcher rather than psychologist to avoid sliding into a therapeutic relationship). This reflexive endeavour or self-critique [42,51] allows for improving the quality and rigor of the data collection: avoidance of premature interpretation or inappropriate probing, fewer assumptions and accentuated sense of curiosity during interviews [25].

Through transcripts of recorded interview data or notes taken during interviews, the researcher may appraise his/her questions and interviewing style and addresses whether the questions asked did not work or forced the answer. Pilot or mock interviews help test the interview guide in achieving the stated objectives and give the opportunity for revision and rethinking the approach and perhaps even, the objectives themselves. In addition, they allow the interviewer to rehearse and to address problematic issues that may arise.

Finally, the interviewer should demonstrate openness, simplicity and sensitivity in exploring sensible concept like disease, death, sexuality. Thus qualitative research interviewing with ill persons requires the affective skills to regulate one’s emotions.

Table 4 illustrates inappropriate verbal interactions in qualitative research interviews.

**Table 4:** Inappropriate verbal interactions for open-ended interviewing

**Type of inappropriate verbal interactions**	**Definitions**
Closed-ended questions	The question invites a yes or no answer and so prevents from elaborating one’s ideas. However, these can be useful to avoid bias, get reticent people to respond and if followed up with open questions. For example: Do you have X? If yes, can you tell me more about that?
Leading questions	The question implies the desired answer and so may respond to the interviewer’s expectations and not reflect the interviewee’s authentic experience.
	E.g., ‘So, your pain bothers you, right?’
Inappropriate probing	A comment or question expressed too early, before the interviewee has completely expressed his/her ideas.
Breaking the silence too early	The interviewer does not recognise the interviewee’s need to reflect before providing his/her answer; the interviewer is ill at ease during silence.
Rushing in questioning	Questions are asked too rapidly which prevents elaboration of the interviewee’s ideas.
Premature interpretation	A comment or interpretation is provided too early which is meant to express understanding, but might bias the patient or may reveal itself to be inappropriate, which may impact on the relationship between interviewer and interviewee.

### Ethical considerations

Interviews must be performed keeping in mind that we address subjects and not *research objects*. Interviews take place in a personal and interpersonal context [18]. The questions asked must not trouble or affect the well-being or development of the interviewee. Before starting the interview, the aims and procedures of the research need to be clearly explained so as the specific role of the interviewer and interviewee. The interviewee has to be informed of the need for their collaboration although, in the research context, they should not expect return (e.g., psychological help) from their participation. The researcher should clearly inform on his/her research (not clinical) role.

Providing the interviewee the choice of a male or female interviewer, particularly for certain cultures and conditions may also be important.

Qualitative interviews must be performed by qualified researchers or by researchers supervised by qualified researchers. Indeed interviews are performed with vulnerable individuals who may be even more destabilized by the evocation of their disease experience. Interviewers must be able to recognise and respect the patient’s need for denial or avoidance. As the interview proceeds, questions should be asked with tact and distinction with respect for the individual’s vulnerability. This is particularly true considering that interviews performed to develop PRO measures are performed with individuals confronted with a disease or with whom specific sensitive topics may be raised (e.g., psychological troubles, sexual life). The interview situation in itself may be threatening; the interviewee may fear losing face, facing one’s contradictions which may undermine his/her self-esteem. In certain contexts, the researcher must admit that interviews may not be possible, e.g., with patients in an advanced stage of disease.

The study protocol aimed at developing a PRO instrument should include submission to a clinical research and ethics committee. Informed consent forms should include that patients are being asked to participate in a research study; the research objective and procedure, the possible disadvantages, risks or benefits of taking part; and that participation is voluntary and that patients have the right to withdraw. In addition, the informed consent form should ensure patients that the information they provide for the study will remain confidential, i.e., no name or personal information will appear on transcribed data and recorded data will be destroyed after the study is completed. Exception to the confidentiality should be made, however, to the patient’s report of adverse medical events or his/her account of self- or other-endangering intentions. These exceptions should be stated with the patient consent and/or assent form.

## Conclusions

The initial steps of developing PROs are based on qualitative research aimed at ensuring the content validity of the instrument being designed. The purpose is to explore and highlight the individual’s experience when confronted with a disease and its treatment, including his/her thoughts, feelings, perceptions, sensations, and attitudes. To access this patient’s experience, the appropriate method of data collection is the interview which draws heavily on interpersonal and communication skills. The interviewer is supposed to create a safe atmosphere allowing the interviewee/group participant to freely and spontaneously express his/her ideas concerning the topic of interest. Specific training and quality assurance initiatives should be implemented to ensure methodological rigour. Interview recording, interviewer training, supervision and a reflexive attitude should be ensured. Interviewing for the development of PROs is performed in a context of illness and vulnerability, and so it is all the more important that, in addition to soliciting informed consent, respectful interactions be ensured throughout the interview process.

## Competing interests

The authors declare that they have no competing interests.

## Authors’ contributions

All authors shared their experience in qualitative research. AB was involved in drafting the manuscript. All authors revised it critically for important intellectual content; and gave final approval of the version to be published.

## Authors’ information

All authors have experience in conducting qualitative interviews for developing PRO measures; in addition AB has clinical experience in psycho-oncology.
